# Comparison of healthcare workers and non-healthcare workers in terms of obsessive-compulsive and depressive symptoms during COVID-19 pandemic: a longitudinal case-controlled study

**DOI:** 10.3389/fpubh.2023.1283317

**Published:** 2023-12-13

**Authors:** Betul Uyar, Suleyman Donmezdil

**Affiliations:** ^1^Department of Psychiatry, Faculty of Medicine, Dicle University, Diyarbakır, Türkiye; ^2^Department of Psychology, Faculty of Literature, Mardin Artuklu University, Mardin, Türkiye

**Keywords:** healthcare workers, obsessive-compulsive symptoms, depressive symptoms, pandemic, COVID-19

## Abstract

**Objective:**

The aim of this study was to investigate the obsessive-compulsive and depressive symptoms of healthcare workers in a case-control setting as longitudinal.

**Method:**

In this study included 49 healthcare workers and 47 non-health workers. A sociodemographic data form, the Maudsley Obsessive-Compulsive Inventory (MOCI), the Symptom Checklist-90 (SCL-90), and the Hamilton Depression Rating Scale (HAM-D) were used to assess individuals between June 1, 2020 and June 30, 2021. We assessed the same healthcare workers after 12 months on June 30, 2021 using MOCI, HAM-D, and SCL-90.

**Results:**

MOCI and SCL-90 obsessive-compulsive subscale scores were significantly higher in the healthcare workers than in the non-health workers. When we assessed MOCI, HAM-D, and SCL-90 obsessive-compulsive subscale scores after 12 months, there was a statistically significant decrease in the scores of all three scales among the healthcare workers.

**Conclusion:**

The results of the study showed that healthcare workers were more likely to have obsessive-compulsive symptoms than non-health workers in the early part of the pandemic on June 1, 2020, as shown by their scores on MOCI and the obsessive-compulsive subscale of SCL-90. When we assessed the same participants after 12 months (June 30, 2021), both MOCI and SCL-90 obsessive-compulsive subscale scores had decreased significantly. In contrast to these results, HAM-D scores significantly increased.

## Introduction

The novel coronavirus disease 2019 (COVID-19) outbreak started in Wuhan, China and has become a serious public health problem that spread worldwide. It was declared as a “pandemic” by the World Health Organization (WHO) in March 2020 (1). Following this, restrictions began in education, social settings, business, and almost every other aspect of life within the framework of quarantine and isolation measures around the world (2, 3). The COVID-19 pandemic has affected every individual to some extent, and biopsychosocial problems have been experienced (4–7). Healthcare workers have been exposed to increased pressure and workload while they also fought in the frontlines during the pandemic period. These workers have faced many physical and psychiatric problems (8–10). Many studies have been conducted to compare different characteristics of healthcare workers to non-health workers during the COVID-19 pandemic. High rates of psychiatric problems such as depression, anxiety, insomnia, and obsessive-compulsive symptoms were found in previous studies (11–15). Similar effects for healthcare workers were observed in studies assessing the effects of past outbreaks (16, 17). In a study by Lu et al. involving 2,042 medical staff and 257 administrative staff, the Hamilton Anxiety and Depression Rating Scales were administered to the participants in February 2020 in China. The mean anxiety and depression scores were significantly higher in medical staff compared to administrative staff (15).

According to a study that included 927 healthcare workers (680 physicians and 247 nurses) in comparison to 1,255 non-health workers, healthcare workers had a higher prevalence of severe insomnia, anxiety, depression, and obsessive-compulsive symptoms than non-health workers in the COVID-19 pandemic period (14). In the same study, for healthcare workers, female sex, being at risk of contact with COVID-19 patients in hospitals, and having organic diseases were associated with anxiety risk. Female sex and having organic diseases were also associated with depression risk. Being at risk of contact with COVID-19 patients in hospitals and having organic diseases were risk factors for obsessive-compulsive symptoms (14). Differently, in a study conducted in Canada with a large sample, non-health workers were found more likely to have anxiety disorder and depression than healthcare workers during the pandemic. Individuals were asked about compulsive handwashing behaviors and worrying about dirt, germs, and viruses. Healthcare workers reported higher rates before and during the pandemic, while non-health workers reported higher rates only during the pandemic (18).

Two studies during the COVID-19 pandemic showed that obsessive-compulsive symptoms started to appear in individuals who did not have OCD (obsessive-compulsive disorder) before or those with OCD who had exacerbations. Fontenelle and Miguel (19) and Rivera and Carballea (20) concluded that the preventive measures recommended by WHO increase the cleaning and washing compulsions of individuals with OCD. In a study conducted with 6,041 participants in the general population, 60.3% of the participants stated that OCD symptoms started, and 53.8% of these participants developed handwashing compulsions (21). Although the increasing rates of depressive and obsessive-compulsive symptoms in healthcare workers in the COVID-19 pandemic are known, because of the high demand for healthcare labor worldwide and the continuation of the destructive effects of the pandemic, preventive and reformative interventions are not on the desired level yet. Studies where the long-term status of obsessive-compulsive and depressive symptoms that started in the first months of the COVID-19 pandemic was investigated were not encountered in the literature. The aim of this study was to investigate the obsessive-compulsive and depressive symptoms of healthcare workers in a case-control setting and reexamine the participants in terms of same symptoms 1 year later.

## Materials and methods

### Research hypotheses

*H_0_:* The COVID-19 pandemic has no effect on obsessive-compulsive behaviors and/or depressive symptoms in healthcare workers.

*H_1_:* The COVID-19 pandemic triggered obsessive-compulsive behaviors and/or caused depressive symptoms in healthcare workers.

### Design and participants

The study was conducted with the participation of nurses and specialist doctors working at the COVID-19 clinic of a research and training hospital in easter Turkey (Gazi Yaşargil Research and Training Hospital, Diyarbakır) and public servants working at institutions other than healthcare institutions. While selecting the participants, based on the power analysis that was conducted at a 0.01 margin of error, 0.08 effect size and in a 95% confidence interval, it was decided that 36 patients in each group and 72 patients in total would constitute the case and control groups. The case group consisted of 49 healthcare workers (12 physicians, 37 nurses) working at the COVID-19 clinic, and the control group consisted of 47 administrative personnel (21 secretarial staff, 6 clinical support staff, 20 cleaning staff). All healthcare workers and non-healthcare workers who were actively working on the data collection dates agreed to participate in the sample. Since there was no one left open, no sampling method was used. The participants in both groups provided informed consent. The study excluded individuals who were on leave, on sick leave, or not working actively in the pandemic period for any reason. Individuals who had been diagnosed with any psychiatric condition before the emergence of the pandemic were also excluded. The data of the study were collected by the researchers using the face-to-face interview method in the period between June 1, 2020 and June 30, 2021. Questions were asked to the participants by the researchers, and the answers given by the participants were recorded on the questionnaire forms.

### Data collection

A sociodemographic data form, the Maudsley Obsessive-Compulsive Inventory (MOCI), the Symptom Checklist-90 (SCL-90), and the Hamilton Depression Rating Scale (HAM-D) were used to assess the participants in June 2020. We assessed the same healthcare workers after 12 months on June 30, 2021 using MOCI, HAM-D, and SCL-90. Information on the data collection forms is given below.

### Sociodemographic data form

It was created by the clinician and used to record data such as age, sex, occupation, and marital status.

### Maudsley obsessive-compulsive inventory

MOCI is a self-report scale that is used to investigate the types and prevalence of obsessive-compulsive symptoms in OCD patients and healthy individuals. The original version of the scale was developed by Hodgson and Rachman (22). The scale that includes true/false statements has four subscales, namely cleaning, checking, doubting, and slowness. The seven-item rumination subscale of the Minnesota Multiphasic Personality Inventory (MMPI) was added to the Turkish adaptation of MOCI. The Turkish version of the scale has been shown to have sufficient validity and reliability (23).

### Symptom checklist-90-R

SCR-90-R, whose initial name was “the Hopkins Symptom Checklist (HSCL-90),” followed by its later name “the Derogatis Symptom Checklist” (24), was finally revised to be named “the Symptom Checklist-90-Revised (SCL-90-R),” and SCL-90-R was translated into Turkish by Dağ (25). The self-report scale that does not include any inversely scored items is a 90-item, 5-point Likert-type scale in which each item is scored between 0 (Not at all) and 4 (Extremely). Using the scale, the Global Symptom Index (GSI) that is scored in the range of 0–4, the Positive Symptom Total (PST) that is scored in the range of 0–90, and the Positive Symptom Distress Index (PSDI) that is scored in the range of 0–4 can be determined.

### Hamilton depression rating scale

HAM-D is a 17-item psychometric instrument that is used in identifying the severity depression in adults (26). The number of items in the scale was lowered to 13. Its maximum score is 53, and higher scores are interpreted as higher severity of depression. Scores of 0–7 indicate no depression, 8–15 indicate mild depression, 16–28 indicate moderate depression, and 29 or higher indicate severe depression (27, 28). The Turkish scale was tested and validated by Akdemir et al. (28).

### Ethical considerations

Ethics committee approval for the study was obtained from Diyarbakır Gazi Yaşargil Research and Training Hospital’s Non-Invasive Clinical Research Ethics Committee (Date: 28.04.2020, Number: 455). In line with the Declaration of Helsinki, the participants were given information about the study, and one of the researchers read the Informed Consent Form to the participants. The participants were included in the study after they gave verbal and written consent.

### Statistical analysis

The data were coded by the researchers. The Statistical Package for the Social Sciences (SPSS) 25.0 IBM statistics program was used to analyze the data. In the analyses, first, descriptive statistics were calculated. In the Shapiro–Wilk test conducted before the analyses, it was determined that the data were non-normally distributed. Homogeneity between the case and control groups was tested using Chi-squared tests and one-way analysis of variance (ANOVA). Differences between mean scores were identified using independent-samples *t*-tests and paired-samples *t*-tests. To identify the sources of significant differences, *post hoc* analyses were carried out. The results were interpreted in a 95% confidence interval. The level of statistical significance was taken as *p* < 0.05.

## Results

Table 1 includes the sociodemographic characteristics of the case and control groups and the results of the homogeneity tests between the groups. The mean age of the healthcare workers was 36.35 ± 5.90, 57.1% of them were male, and 75.5% were single. Additionally, 61.2% of them lived with their family, 85.7% had no family history of psychiatric illness, and 91.8% had no personal history of psychiatric illness. The mean age of the non-health workers was 37.11 ± 6.74, 55.3% of them were women, 83% were single, 91.5% were living with their families, and 89.4% had no history of psychiatric illness. There was no statistically significant difference between the two groups regarding their age, sex, marital status, living arrangements before the onset of the pandemic, personal history of psychiatric illness, or family history of psychiatric illness. The mean number of years of education in the case group was significantly higher than that in the control group. The rate of living alone during the pandemic period was significantly higher in the case group than in the control group (Table 1).

**Table 1:** Comparison of some sociodemographic and clinical characteristics of healthcare workers and non-health workers (*N* = 96).

Items	Healthcare workers (*n* = 49)	Non-health workers (*n* = 47)	Homogeneity test between two groups
	Mean ± SD	Mean ± SD	
**Age**	36.35 ± 5,90	37.11 ± 6.74	*F* = 0.131
			*p* = 0.558
**Education time (years)**	15.94 ± 1.30	15.09 ± 0.41	*F* = 0.336
			*p* = 0.031*
	***n* (%)**	***n* (%)**	
**Sex**			
Female	21 (42.9)	26 (55.3)	χ^2^ = 0.314
Male	28 (57.1)	21 (44.7)	*p* = 0.225
**Marital status**			
Single	37 (75.5)	39 (83)	χ^2^ = 1.231
Married	12 (24.5)	8 (17)	*p* = 0.37
**Living (before the pandemic)**			
With family	43 (87.8)	40 (85.1)	χ^2^ = 1.711
Alone	6 (12.2)	7 (14.9)	*p* = 0.706
**Living (during the pandemic)**			
With family	30 (61.2)	43 (91.5)	χ^2^ = 2.102
Alone	19 (38.8)	4 (8.5)	*p* = 0.691
**History of psychiatric illness**			
Yes	4 (8.2)	5 (10.6)	χ^2^ = 1.148
No	45 (91.8)	42 (89.4)	*p* = 0.679
**Family history of psychiatric illness**			
Yes	7 (14.3)	8 (17)	χ^2^ = 2.132
No	42 (85.7)	39 (83)	*p* = 0.514

*p < 0.05; χ^2^, Chi-squared test; *F*, one-way analysis of variance (ANOVA); SD, standard deviation. Bold values with statistical significance (*p* < 0.01, *p* < 0.05).

There was a statistically significant difference between the living arrangements of the case group before and after the pandemic, while there was no significant difference in the control group. According to the results of the Kruskal-Wallis test, 30.6% of the participants in the case group started to live alone during the pandemic, while they used to live with their family before the pandemic (Table 2).

**Table 2:** Change in residence of individuals before and at the beginning of the pandemic (*N* = 96).

	Healthcare workers (*n* = 49)	Non-health workers (*n* = 47)
	*n* (%)	*n* (%)
Family➔Alone for isolation	15 (30.6)	–
Alone➔Family	2 (4.1)	3 (6.4)
No change	32 (65.3)	44 (93.6)
Test and *p*-value	*t* = 0.614	*t* = 0.548
	*p* = 0.002**	*p* = 0.083

*t*, independent-samples *t*-test; **p < 0.001. Bold values with statistical significance (*p* < 0.01, *p* < 0.05).

The MOCI scores and the SCL-90 obsessive-compulsive subscale scores of the case group were significantly higher than those of the control group (Table 3).

**Table 3:** Comparison of scale scores of healthcare workers and non-health workers (*N* = 96).

Scales	Healthcare workers (*n* = 49)	Non-health workers (*n* = 47)	Test and *p*-value
	Mean ± SD	Mean ± SD	
MOCI	11.69 ± 6.46	8.79 ± 3.83	*t* = 2.134
			*p* = 0.009**
HAM-D	7.06 ± 4.65	7.02 ± 4.36	*t* = 1.612
			*p* = 0.965
**SCL-90-R**			
Somatization	0.62 ± 0.32	0.65 ± 0.28	*t* = 1.321
			*p* = 0.667
Interpersonal sensitivity	0.58 ± 0.30	0.58 ± 0.28	*t* = 0.516
			*p* = 0.928
Anxiety	0.73 ± 0.53	0.71 ± 0.48	*t* = 0.312
			*p* = 0.816
Phobia	0.54 ± 0.27	0.55 ± 0.27	*t* = 0.213
			*p* = 0.881
Depression	0.65 ± 0.37	0.63 ± 0.39	*t* = 0.711
			*p* = 0.807
Hostility	0.56 ± 0.29	0.56 ± 0.30	*t* = 0.819
			*p* = 0.939
Paranoid ideation	0.54 ± 0.26	0.56 ± 0.28	*t* = 1.112
			*p* = 0.759
Psychoticism	0.54 ± 0.23	0.54 ± 0.23	*t* = 811
			*p* = 0.851
Obsessive-compulsive	1.16 ± 0.50	0.87 ± 0.38	*t* = 2.312
			*p* = 0.002**

*t*, independent-samples *t*-test; **p < 0.01. Bold values with statistical significance (*p* < 0.01, *p* < 0.05).

In the assessments of the MOCI, HAM-D, and SCL-90 obsessive-compulsive subscale scores after 12 months, there was a statistically significant decrease in all three scale scores among the participants in the case group (Table 4).

**Table 4:** Comparison of scale scores of healthcare workers in 2020 and 2021 (*N* = 96).

Healthcare workers (*N* = 49)	First measurement	Second measurement	Test and *p*-value
Scales	Mean ± SD	Mean ± SD	
MOCI	11.69 ± 6.46	8.82 ± 3.84	*t* = 0.258
			*p* = 0.001**
HAM-D	7.06 ± 4.65	8.96 ± 5.10	*t* = 0.311
			*p* = 0.040*
Obsessive-compulsive subscale of SCL-90-R	1.16 ± 0.50	0.80 ± 0.60	*t* = 0.123
			*p* = 001**

*t*, paired-samples *t*-test; **p < 0.01; *p < 0.05. Bold values with statistical significance (*p* < 0.01, *p* < 0.05).

## Discussion

The results of the study explain the obsessive-compulsive and depressive symptoms of healthcare workers during the COVID-19 pandemic. According to the results of the homogeneity test, there was no statistically significant difference between the case group including healthcare workers and the control group including non-health workers in terms of their age, sex, marital status, living arrangements before the pandemic, personal history of psychiatric illness, or family history of psychiatric illness. The mean duration of education among the healthcare workers (case group) was significantly longer than that in the non-health workers (control group). Since the healthcare workers in our study consisted of specialist doctors and nurses, it was expected that the education duration of the healthcare workers would be longer than that of the non-health workers. The rate of living alone during the pandemic was significantly higher in the case group than in the control group. It was found that 30.6% of the participants in the case group had left their families for isolation to protect them from exposure to the virus during the pandemic. In a study with a broad sample by Lu et al., 22% of medical staff and 14.4% of administrative staff answered yes to the statement “I feel lonely being isolated from my loved ones,” and the difference between the two groups was statistically significant (25).

Previous studies have reported that healthcare workers fighting COVID-19 are exposed to acute stress and therefore experience post-traumatic stress disorder and depression, and become mentally and physically weakened (29–31). Several studies on the mental health of healthcare workers during the COVID-19 pandemic revealed higher levels of stress, anxiety, depressive symptoms, and obsessive-compulsive symptoms (14, 32–34). Zhang et al. found that healthcare workers had a higher prevalence of severe insomnia, anxiety, depression, and obsessive-compulsive symptoms than non-health workers during the COVID-19 pandemic (14). In another study, the mean levels of anxiety and depression determined using the Hamilton Anxiety and Depression Rating Scales were significantly higher in medical staff compared to administrative staff (15). Unlike other studies, Mrklas et al. determined that non-health workers were more likely to have anxiety disorder and depression than healthcare workers. Individuals were asked about compulsive handwashing and worrying about dirt, germs, and viruses. Healthcare workers reported higher rates both before and during the pandemic, while non-health workers reported higher rates only during the pandemic (18). Consistent with this study, Wang et al. reported that early in the COVID-19 pandemic, the general public adopted handwashing after touching contaminated objects, washing their hands with soap, and always washing their hands after coughing, sneezing, or rubbing their nose as new habits (35). Based on our findings, the current effects of this public health problem experienced by healthcare professionals in the short and long term should be determined urgently. Intervention is needed to counteract these effects and rebuild the health of these individuals. The primary aim of such an intervention would be to support the mental health of healthcare professionals.

In this study, MOCI scores and SCL-90 obsessive-compulsive subscale scores were significantly higher in the case group than in the control group. There was no significant difference between the groups in terms of their scores in HAM-D and the other subscales of SCL-90. The higher obsessive-compulsive scale scores in the healthcare workers in this study can be explained by the fact that the study was conducted in the main pandemic hospital in a large city and a central region in Turkey. The fact that the novel coronavirus 2019 (SARS-CoV-2) is a newly encountered and poorly known virus, and there is a lack of adequate information about COVID-19, the participants working in the pandemic hospital were worried about their own health and that of their families, and their lack of social support may also have contributed to their high obsessive-compulsive scale scores.

In the assessments of MOCI, HAM-D, and SCL-90 obsessive-compulsive subscale scores after 12 months in our study, there was a statistically significant decrease in MOCI and SCL-90 obsessive-compulsive subscale scores and a significant increase in HAM-D scores. There are not enough longitudinal studies related to this subject in the literature. Loosen et al. assessed depressive and obsessive-compulsive symptoms in the United Kingdom for the general population two times, in April and August 2020 (36). They found an increase in obsessive-compulsive symptoms both at the beginning of the pandemic in April and in the following assessments in August. For depressive symptoms, there was an increase at the beginning of the pandemic, but in the second assessment, depressive symptoms had decreased. Previous studies also emphasized that they found the same increase in OC symptoms when these symptoms were only measured using pandemic-irrelevant items, suggesting that the observed increase in OC symptoms throughout the lockdown cannot be attributed to just adaptive protective behaviors during the pandemic (36, 37). In the study, depressive symptoms may have decreased due to adaptation development. The finding that obsessive-compulsive symptoms did not decrease can be explained by the early second evaluation in the aforementioned study. In our study, the second evaluation was made in July 2021. Exposure and response prevention are proven cognitive behavioral therapy interventions for obsessive-compulsive disorder (38). Due to the fact that the participants were working in a pandemic hospital, the prolongation of their exposure and the decrease in their possibility of avoidance may explain the decrease in the severity of their obsessive-compulsive symptoms over time. Likewise, the discovery of vaccines for COVID-19 and the fact that healthcare professionals in Turkey had started to be vaccinated may have contributed to the decrease in obsessive-compulsive symptoms.

### Limitations and strengths

This study had several limitations. The fact that the second evaluation was made after 12 months and the prolongation of the period of pandemic measures may have caused an increase in depressive symptoms in healthcare professionals due to feelings of burnout and hopelessness, as well as the uncertainty of the process. The small sample size may have resulted in the insignificance of the statistical differences between the case and control groups. The fact that the second evaluation was made only in healthcare workers was another limitation. However, making the first evaluation in June 2020, in the early stages of the pandemic, and re-evaluating the healthcare workers 1 year later were strengths of our study. The scale used in our study includes expert evaluation. The participants’ subjective evaluations were not included in our findings, which demonstrates the strength of our findings.

## Conclusion

Our results showed that healthcare workers were more likely to have obsessive-compulsive symptoms than non-health workers, as shown by their MOCI and SCL-90 obsessive-compulsive subscale scores in the early part of the pandemic in June 2020. When we assessed the healthcare workers after 12 months (July 2021), both MOCI and SCL-90 obsessive-compulsive subscale scores had decreased significantly. In contrast to these results, the HAM-D scores of the participants increased significantly. Our study contributes to the literature in terms of being a longitudinal follow-up study and showing that obsessive-compulsive symptoms decreased, and depressive symptoms increased in healthcare workers after 12 months during the pandemic period. Healthcare workers constitute a special group because they work in difficult conditions under high stress and danger in all periods and especially during periods of health crises such as epidemics or pandemics. In this context, experimental studies should be conducted with the participation of healthcare workers. The working conditions of healthcare workers should be improved, and they should be supported more by the state in social terms. Initiatives and research that will increase the psychological resilience of healthcare professionals should be planned.

## Data availability statement

The original contributions presented in the study are included in the article/supplementary material, further inquiries can be directed to the corresponding author.

## Ethics statement

The studies involving humans were approved by Ethics Committee approval for the study was obtained from Diyarbakır Gazi Yaşargil Research and Training Hospital’s Non-Invasive Clinical Research Ethics Committee (Date: 28.04.2020, Number: 455). In line with the Declaration of Helsinki, the participants were given information about the study, and one of the researchers read the Informed Consent Form to the participants. The participants were included in the study after they gave verbal and written consent. The studies were conducted in accordance with the local legislation and institutional requirements. The participants provided their written informed consent to participate in this study. Written informed consent was obtained from the individual(s) for the publication of any potentially identifiable images or data included in this article.

## Author contributions

BU: Conceptualization, Data curation, Funding acquisition, Investigation, Methodology, Project administration, Software, Writing – review & editing. SD: Conceptualization, Data curation, Formal analysis, Funding acquisition, Methodology, Validation, Writing – original draft.
